# A New Perspective on Diabetic Foot Syndrome: From Caregiver to Caregiving—The Role of Clinical Complexity

**DOI:** 10.3390/jpm16100499

**Published:** 2026-09-26

**Authors:** Mauro Maurantonio, Lorenzo Rubrigi, Claudia Castellano, Riccardo Cuoghi Costantini, Davide Valle, Paolo Ventura

**Affiliations:** 1 Department of Internal Medicine, General and Post-Acute, University Hospital of Modena, University of Modena and Reggio Emilia, 41125 Modena, Italy; lorenzo.rubrigi@ausl.re.it (L.R.); davide.valle@ausl.re.it (D.V.); paoloven@unimore.it (P.V.); 2 Hemodialysis Center of Sassuolo, AUSL Modena, 41049 Modena, Italy; claudia-castellano@tiscali.it; 3 Department of Medical and Surgical Sciences for Mother, Child and Adult, University of Modena and Reggio Emilia, 41125 Modena, Italy; riccardo.cuoghicostantini@unimore.it

**Keywords:** diabetic foot ulcer, multimorbidity, caregiver

## Abstract

**Background/Objectives**: Diabetic foot syndrome (DFS) is a serious complication of diabetes that poses a high risk of morbidity/mortality. The management of these patients requires a multidisciplinary team approach, as these patients are “complex” and “fragile”. Despite this, the rate of re-amputation remains high. This study aims to evaluate the degree of multidimensional complexity in patients with DFS using specific tools and to determine if it is an additional risk factor for amputation. **Methods**: The study was conducted on a cohort of 115 patients referred to the Tertiary Center of the University Hospital of Modena from January to December 2021. The study used 12 variables to assess the patients, including age, sex, caregiver presence, Barthel Index, Lawton–Brody Scale, Mini-Mental State Examination, Cumulative Illness Rating Scale (CIRS), SINBAD system, Malnutrition Universal Screening Tool and the Italian National Association of Social Workers Tool. All patients were taken care of by a multidisciplinary team in order to standardize care according to updated guidelines. Patients were classified based on amputation and revascularization status. Statistical analyses were performed using the R software version 4.1.1. **Results**: The analysis showed that a greater clinical complexity and a more severe ulcer had a greater risk of amputation (respectively, OR 3.13, *p* 0.035 and OR 1.53, *p* 0.015). The study confirms the predictive value of tools such as CIRS and SINBAD in amputation risk stratification for patients with DFS. **Conclusions**: Although this study has limitations due to the small sample size, the use of a multidisciplinary approach and multidimensional evaluation tools can identify “unconventional” risk factors that, if modified, could delay and/or reduce the risk of amputation. Further studies are needed to investigate the role of caregiving in the prevention of amputation in patients with DFS.

## 1. Introduction

Diabetes mellitus is a high-prevalence chronic disease with an increasing trend and a high impact on health systems: it is estimated that 61 million people are affected in Europe [1], of which 4 million are in Italy [2]. Diabetic subjects have a more than double risk of hospitalization (235 against 99 per 1000 people) and longer average hospitalization periods (11.3 days against 9.9) compared to non-diabetics, with higher costs associated with complications and comorbidities [3]. In a diabetic patient, the presence of multimorbidity worsens the prognosis: in about 80% of cases, there is at least one other chronic disease, such as COPD, ischemic cardiomyopathy, heart failure, chronic kidney disease, and non-alcoholic fatty liver disease, with a significant increase in mortality for all causes, especially cardiovascular ones (OR 1.22–5.46) [4,5,6].

Diabetic foot syndrome (DFS) is defined by the presence of an ulcer with varying degrees of ischemia, infection, and/or neuropathy in a diabetic individual [7]. It represents a formidable complication of diabetes mellitus, characterized by high mortality, morbidity and commitment of resources. The lifetime risk of a person with diabetes to develop a foot ulcer is 15–25% [8]; every 30 s, in the world, a lower limb is amputated due to diabetes [9], and the probability of ulcer recurrence is 40% within 12 months [10].

A patient with diabetic foot syndrome can be defined as “complex” and “fragile”, with a 4-times higher risk of cardiovascular mortality than diabetics without ulcers; it is known that a patient with a previous amputation has a 5-year mortality risk of 39–68% [11].

Among the main risk factors for amputation in DFS are the presence of PAD (Peripheral Arterial Disease), peripheral neuropathy, a concurrent infection, malnutrition, and renal disease, especially in the presence of comorbidities [12,13,14]. Even the lack of therapeutic education of patients and their caregivers seems to interfere with ulcer healing [15], even if, to date, validated systems to evaluate the adequacy of caregiving in patients with DFS do not exist. The impact of DFS is high in terms of economic resources on Welfare Systems in the World and worsens the patient’s and his caregivers’ quality of life [16]; for these reasons, a patient affected by DFS can be defined as “complex” and “fragile”. International guidelines suggest that management by a multidisciplinary team appears to be able to reduce major amputations and alleviate the burden in DFS [16]. Despite this, the rate of re-amputations continues to be high (19% within 12 months and 37% within 5 years) [17]; this could be related to risk factors not yet identified.

### Complexity in DFS

In the literature, there are different definitions of “frailty”, a condition associated not only with advanced age but, above all, with bio-psycho-social “complexity” [18,19].

It is defined as “a clinically recognizable state of increased vulnerability resulting from aging-associated decline in reserve and function across multiple physiologic systems such that the ability to cope with everyday or acute stressors is compromised” [20] or as an “impaired resolution to homoeostasis following a stressor event” [21]. Overall, frailty is associated with a more than doubled risk of mortality in men and women aged 45 years and older, increases the risk of hospitalization (OR 1.82), and results in loss of basic autonomy (OR 2.05) [22]. The risk increases in the presence of multimorbidity [21], outlining a “complex” patient phenotype.

The “complex” phenotype is a peculiar identity that does not result from the mere sum of the individual pathologies that compose it but from their multidimensional interaction. Furthermore, it includes biological, socioeconomic, cultural, behavioral and environmental characteristics as key determinants of patient health [23] (Table 1).

The fragile and complex patient requires a global and integrated approach (bio-psycho-social approach) [18] and the use of tools capable of assessing multidimensional health that can be adapted to the specific pathology [27,28,29,30]. Currently, there are no validated tools capable of facilitating the multidimensional assessment of a patient with DFS.

The aim of our study is to describe, through specific tools, the degree of multidimensional complexity in a sample of patients with DFS belonging to the 3rd level of the Provincial Diabetic Foot ICP (Integrated Care Pathway) of Modena and to evaluate whether it constitutes an additional risk factor for amputation or not. Secondary outcomes include rate of hospitalization and revascularization procedures.

## 2. Materials and Methods

This retrospective study was conducted on a cohort of patients consecutively referred to the Metabolic Internal Medicine Department of the University Hospital of Modena (Tertiary Center of the Diabetic Foot ICP), both inpatient and outpatient, in the period of January–December 2021. For a multidimensional evaluation, each patient was given specific forms validated in the literature and/or suggested by International Guidelines (Table 2).

The general characteristics of the population examined are described in Table 3.

According to International Guidelines [7], amputation was classified as “minor” if performed at or distal to the ankle, “major” if performed proximal to the ankle, and “absent” if not performed. Revascularization was classified as Percutaneous Transluminal Angioplasty (PTA), “Bypass” of the lower limb, or “no revascularization”.

For each patient, 12 variables were identified, 6 of which were numerical, relating to the score obtained in Barthel Index, Lawton–Brody Scale, MMSE (Mini-Mental State Examination), CIRS (Cumulative Illness Rating Scale), and SINBAD system, and based on age and 6 categorical variables based on sex, on the score obtained in MUST (Malnutrition Universal Screening Tool), the ONAS tool (Ordine Nazionale Assistenti Sociali—Italian National Association of Social Workers), whether or not a revascularization and/or amputation intervention was performed, and the presence or absence of a caregiver.

All patients included in the study belong to the Tertiary Center of the ICP for the treatment of diabetic foot in the province of Modena and were taken care of by a multidisciplinary team made up of internists, vascular surgeons, infectious disease specialists, orthopedists, radiologists, endocrinologists, dermatologists, microbiologists and clinical psychologists in order to standardize care [37].

### Statistical Analysis

All statistical analyses were performed using the R software version 4.1.1.

The *p*-values obtained from the tests performed were considered significant when <0.05. Numerical variables were described as the mean ± standard deviation or as the median and interquartile range, while dichotomous and categorical variables are reported in terms of absolute frequencies and percentages. The association between numerical variables was measured using Pearson’s product–moment correlation, while the degree of association between categorical variables was evaluated using Pearson’s χ^2^ test or Fisher’s exact test. The association analysis between categorical and numeric variables was performed using one-way ANOVA. Odds ratios and related confidence intervals were calculated on the basis of coefficients and variances estimated from logistic regression models. A multivariable logistic analysis was performed to correct for the effect of the variables considered. In particular, all the variables associated with the outcome in the univariable analysis were entered into the model, as well as other variables of clinical interest.

For minor complications, the multivariable model included 7 predictors considered to be of clinical interest (sex, MMSE, Barthel Index, CIRS, SINBAD, caregiver presence and age), resulting in an event per variable ratio equal to 5.4, which is acceptable according to [38].

Observations presenting at least one missing value with respect to the variables of interest were excluded from the analysis.

## 3. Results

### 3.1. Characteristics of the Sample Examined

During the observation period, a total of 115 patients with DFS were identified (84 M, 73%; 31 F, 27%) with a mean age of 70.6 ± 10.6. Of these, 41 (35.7%) were outpatients (mean age, 71 years), while 74 (64.3%) were inpatients (mean age, 70 years). Both outpatients and inpatients were taken care of by the multidisciplinary team (Diabetic-Foot-Care Team) and subjected to standard diagnostic-therapeutic care according to updated guidelines.

Table 4 describes the characteristics of hospitalized patients (group A) and outpatients (group B). It can be seen, from the descriptive analysis of the sample examined, that inpatients (group A) were characterized, compared to outpatients (group B), by worse ulcer status and greater clinical complexity, with a greater risk of malnutrition and greater recurrence to revascularization and amputation procedures. The degree of autonomy was worse in hospitalized patients, both in basic and instrumental activities. The caregiver was found to be more present in hospitalized patients. The cognitive level was comparable between group A and group B. The socioeconomic complexity was also similar (moderate level); however, due to the large amount of missing data it was not possible to evaluate the impact of this last variable on the outcomes, and it was therefore excluded from the statistical analysis.

### 3.2. The Bio-Psycho-Social “Complexity”

By evaluating the sample as a whole, it was also revealed that a higher cognitive level is related to greater autonomy in ADLs (Activities Daily Living) and IADLs (Instrumental Activities Daily Living) and that there is an association between advanced age and a lower MMSE score. Advanced age correlates with lower autonomy in ADLs (*p* 0.002). Multimorbid patients (high CIRS score) are less autonomous in both ADLs and IADLs. Moreover, there is a direct correlation in the degree of autonomy in ADLs and IADLs.

In the sample examined, the presence of the caregiver appears to be more frequent in elderly subjects and in no self-sufficient subjects in ADLs (*p*-values of 0.015 and 0.016, respectively). It seems that many self-sufficient ADL patients have a caregiver: this probably depends on the fact that they are not young patients (mean age of the sample, 70 years), that they may have difficulty in IADLs, and that therefore they still need home assistance.

From our study it is also possible to observe that the male sex is associated with worse autonomy in IADLs compared to the female ones (*p*-value = 0.04; mean difference = −0.17): this may depend on the fact that for cultural reasons, many older males have never performed some of the activities described in IADLs (doing the laundry, housekeeping, and food preparation), and this can alter the final score, as already known in the literature [39].

### 3.3. Outcomes

In our study, 72 patients (62.6% of the total) did not undergo amputation, and 43 patients (37.4% of the total) underwent amputation: 38 patients (33% of the total, 2 outpatients, and 36 inpatients) underwent surgery with minor amputation, and only 5 patients (6.8% of the total) required a major amputation on admission. The prevalence of minor amputation is higher in hospitalized patients (48.6% vs. 4.9%) (Table 3).

During the observation period, revascularization was performed in 86% of the subjects undergoing amputation, in line with guidelines. Only in 6 cases was the amputation not preceded by revascularization during hospitalization: in 5 cases (4 M and 1 F) it was performed in the previous six months, and in 1 case, there were good distal flows on Doppler evaluation. A total of 100% of subjects revascularized via bypass subsequently underwent amputation, compared to 69% of patients subjected to revascularization via PTA, probably due to a greater severity of vascular compromise. All patients undergoing “major amputation” had a high score on the SINBAD system classification (between 5 and 6), and there are no significant differences between the other variables compared to the total sample. Only 5 major amputations occurred in the study population. For this reason, a multivariable model was not estimated for major amputation.

In our study, “minor amputation” appears to be more frequent in males (although this trend is not statistically significant; OR = 2.079, *p*-value 0.134), which is consistent with data from the literature, in which the male sex is considered to be a risk factor for amputation [12].

As far as the assessment of clinical complexity is concerned, in the sample examined, patients with greater clinical complexity (higher CIRS severity index) and a more severe level of ulcer status (higher score on the SINBAD system) have a greater risk of minor amputation: this confirms the predictive value of tools such as CIRS and SINBAD in amputation risk stratification, especially in “fragile” and “complex” patients. This association is significant both in the univariable analysis (respectively, OR 3.5, *p* 0.006 and OR 1.64, *p* 0.001) (Figure 1) and in the multivariable analysis (respectively, OR 3.13, *p* 0.035 and OR 1.53, *p* 0.015). The final multivariable model is displayed in Figure 2, while Table 5 shows the ORs, 95% CIs and *p*-values obtained from this model.

We compared the models with and without CIRS using changes in the AUC, likelihood ratio test and Hosmer–Lemeshow test for assessing calibration. We have reported the obtained results in Table 6.

Particularly suggestive is that, unlike what is described in the literature [40,41], in our sample the presence of a caregiver does not appear to be a protective factor (Figure 2). In the multivariable model for minor amputation, caregiver presence was not independently associated with this outcome (adjusted OR was 0.96, with 95% CI ranging from 0.31 to 2.97). It is probable that an assessment of the mere presence/absence of a caregiver is not sufficient and that a careful assessment of its adequacy is mandatory, in order to be able to take any therapeutic education measures. Further studies will be needed to investigate the role of caregiving in the prevention of major amputation in patients with diabetic foot syndrome.

## 4. Discussion

Our study analyzes the characteristics of a sample of 115 diabetic patients belonging to the Tertiary Center of the ICP for diabetic foot care in the province of Modena (74 inpatients vs. 41 outpatients). In the sample examined, inpatients (group A) appear to present greater clinical complexity (mean CIRS, 2.23) than outpatients (group B; mean CIRS, 1.86), confirming the appropriateness of hospitalization and the advantage of a multidisciplinary approach: only 5 patients underwent major amputation. Our study confirms some risk factors for amputation already known in the literature [42,43] and definable as “classics”, such as the high score obtained on the SINBAD system and male sex. Our study demonstrates that the approach to DFS through a multidisciplinary team that identifies and manages not only the acute complication of the ulcer but also the multimorbidity measured through specific assessment forms is able to reduce major amputations. In particular, the score obtained with the CIRS tool, in addition to correlating with mortality, hospitalization risk, disability and impact of drug therapy [44,45], appears to be independently associated with the risk of amputation since it identifies the degree of complexity of a patient with DFS.

Currently, there are no specific indications for the management of multimorbidity since most guidelines focus on single diseases and are heterogeneous and not very comparable [21,46,47]. A 2018 review by Chiang et al. emphasizes the need for clinical guidelines to support a ‘holistic’ approach to the complex care needs of patients affected by type 2 diabetes with multimorbidity, where care of the whole person should be the primary concern [5].

A diabetic foot is to be considered, to all intents and purposes, just one side of a very complex systemic syndrome, which not only hurts the body in its entirety and occurs as one of the organ damages caused by chronic hyperglycemia (such as cardiovascular, renal, cerebral, and hepatic damage) but also affects the patient’s autonomy and ability to fend for himself. In our study, the presence of a caregiver does not seem to be significantly related to other variables, in particular with the amputation outcome; this is probably motivated by the fact that the mere presence/absence of a caregiver is not sufficient to be associated (directly or inversely) with the “amputation” outcome as it would be necessary to verify its adequacy. In the present retrospective study, the caregiver variable was considered as a categorical variable, distinguishing between the presence and absence of a caregiver. Caregiver presence was determined from the medical history collected during the patient’s clinical assessment. Specifically, a caregiver was considered to be present when the medical history indicated the existence of a person actively involved in one or more aspects of the patient’s daily management, including wound dressing, supervision of medication intake, management of organizational and administrative tasks (e.g., appointment management), and assistance with activities of daily living (ADLs). Therefore, this variable did not simply indicate the presence of a family member or cohabitant but rather the presence of a person who effectively provided care and support to the patient. However, no formal assessment of caregiver adequacy was performed. As already stated in this manuscript, we did not identify any validated tools specifically designed to assess the adequacy of caregiving in patients with diabetic foot syndrome in the literature. Furthermore, as this was a retrospective study conducted on a cohort of patients evaluated between January and December 2021, the “caregiver presence” variable was not prospectively defined according to a dedicated caregiving assessment protocol. This not only represents a limitation of the study but also an important consideration arising from our findings: a simple “presence/absence” classification may not be sufficiently informative to characterize the potential contribution of caregiving to clinical outcomes. Indeed, in our sample, caregiver presence was not independently associated with minor amputation. We agree that both caregiver presence/absence and the potential adequacy of caregiving may be influenced by multiple factors related to the patient and their context, including disease severity, functional status, treatment adherence, socioeconomic circumstances, access to healthcare, and timeliness of care. Some of these factors were assessed in our study. In particular, caregiver presence was associated with certain patient characteristics, including older age and lower functional autonomy. However, given the retrospective nature of the study, these associations do not allow for causal relationships to be established.

Several potential confounders were measured, although not all were incorporated into the final multivariable model. As already mentioned, the multivariable models were constrained due to the low sample size and included just sex, MMSE, Barthel Index, CIRS, SINBAD, caregiver presence and age as covariates. Therefore, potential residual confounding may be present. The Section 4 has been revised to explicitly address this limitation. Additional measured confounders included nutritional status (measured using MUST), socioeconomic status (ONAS scale), treatments and access to specialist care, HbA1c, diabetes duration and renal dysfunction.

It is therefore important to underline the importance of correctly investigating the state of the ulcer, the presence of co-pathologies capable of significantly affecting the patient’s functional autonomy and the need to evaluate the adequacy, not only the presence, of a caregiver through validated and appropriate tools. Since 2005, the WHO has emphasized the role of therapeutic education in diabetic patients and the importance of involving the caregiver as a way to increase adherence to treatment. Adequate caregiving is considered an essential and integral part of foot ulcer prevention, as it is widely thought to be fundamental that people with diabetes that are at-risk of foot ulceration need to protect their feet from external stress and to follow care and support programs. For this reason, personalizing care for diabetics also means involving the caregiver, and this presents a new opportunity to improve outcomes. To reduce amputations, it is essential to take care of all pathologies and train the caregiver in the co-management of the diabetic foot ulcer through sessions of therapeutic education and monitoring progress by intervening before an unfavorable evolution.

## 5. Conclusions

Albeit with some limitations, mainly constituted by the small size of the sample, this analysis on 115 patients with complicated diabetic foot ulcer admitted to the Tertiary Center of Modena showed that in subjects with diabetic foot syndrome undergoing amputation, risk factors are represented by the male gender, ulcer classification, clinical complexity and impairment of functional autonomies in the presence of multimorbidity. In our series, the presence of multimorbidity appears to be an independent risk factor for minor amputation.

In our sample, caregiver presence was not independently associated with minor amputation. Future prospective studies should therefore assess not only caregiver presence but also the intensity, characteristics, and adequacy of caregiving in a structured manner, including involvement in wound care, treatment management, activities of daily living, and organizational aspects of care. Given the complexity of patients with diabetic foot syndrome and the fact that several individuals may simultaneously contribute to different aspects of patient care, our findings led us to consider that it may be more appropriate to assess the overall caregiving provided to the patient, rather than simply the presence or absence of a single caregiver. This could be achieved through the development and subsequent validation of dedicated tools capable of assessing these different dimensions of caregiving in a more detailed and comprehensive manner. Early identification of inadequate or partially adequate caregiving could help identify patients requiring a higher level of support, even before referral to a specialist team. Even when the underlying determinants cannot be directly modified, their identification could allow for care to be tailored to the individual patient through closer community-based monitoring, targeted educational interventions for the patient and their caregiving network, and, when necessary, earlier involvement of a specialist team.

In conclusion, a patient with DFS can be considered “fragile” and “complex” and requires a multidimensional approach: in such patients, the use of specific multidimensional evaluation tools can identify “unconventional” risk factors which, if modified, could delay and/or reduce the risk of amputation. In our experience, with the correct management of a patient with DFS, together with adequate management of diabetic ulcers; the correct choice of medical–surgical therapy; specific training involving health professionals, patients and their caregivers; and the improvement of multidisciplinary pathways, the correction of “conventional” and “unconventional” modifiable risk factors can improve outcomes and must be considered in the Integrated Care Pathway (ICP) aimed at defining a “personalized and holistic” approach.

## Figures and Tables

**Figure 1:**
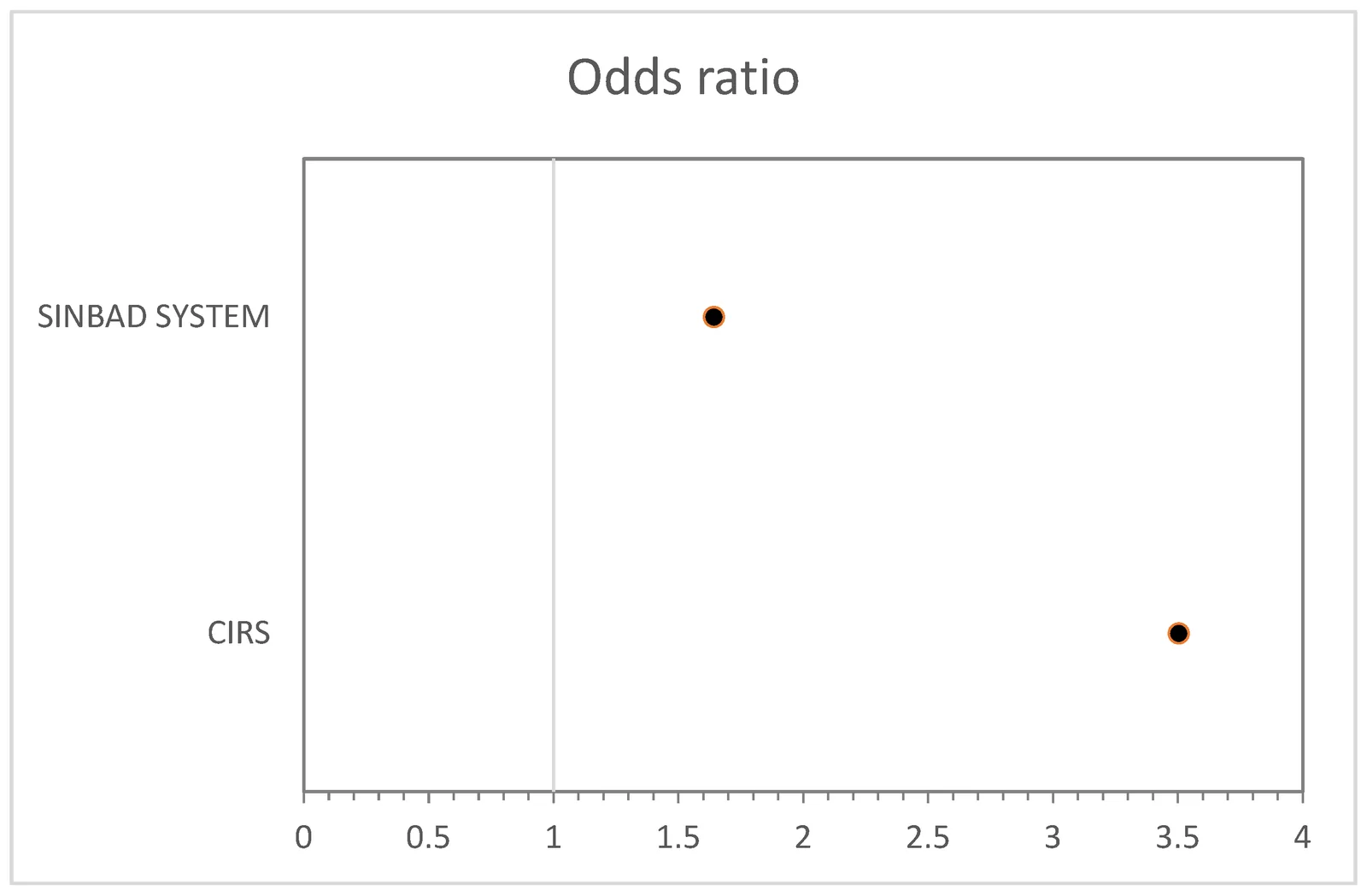
Representation of the ORs and confidence intervals of the univariable model for the “minor amputation” outcome.

**Figure 2:**
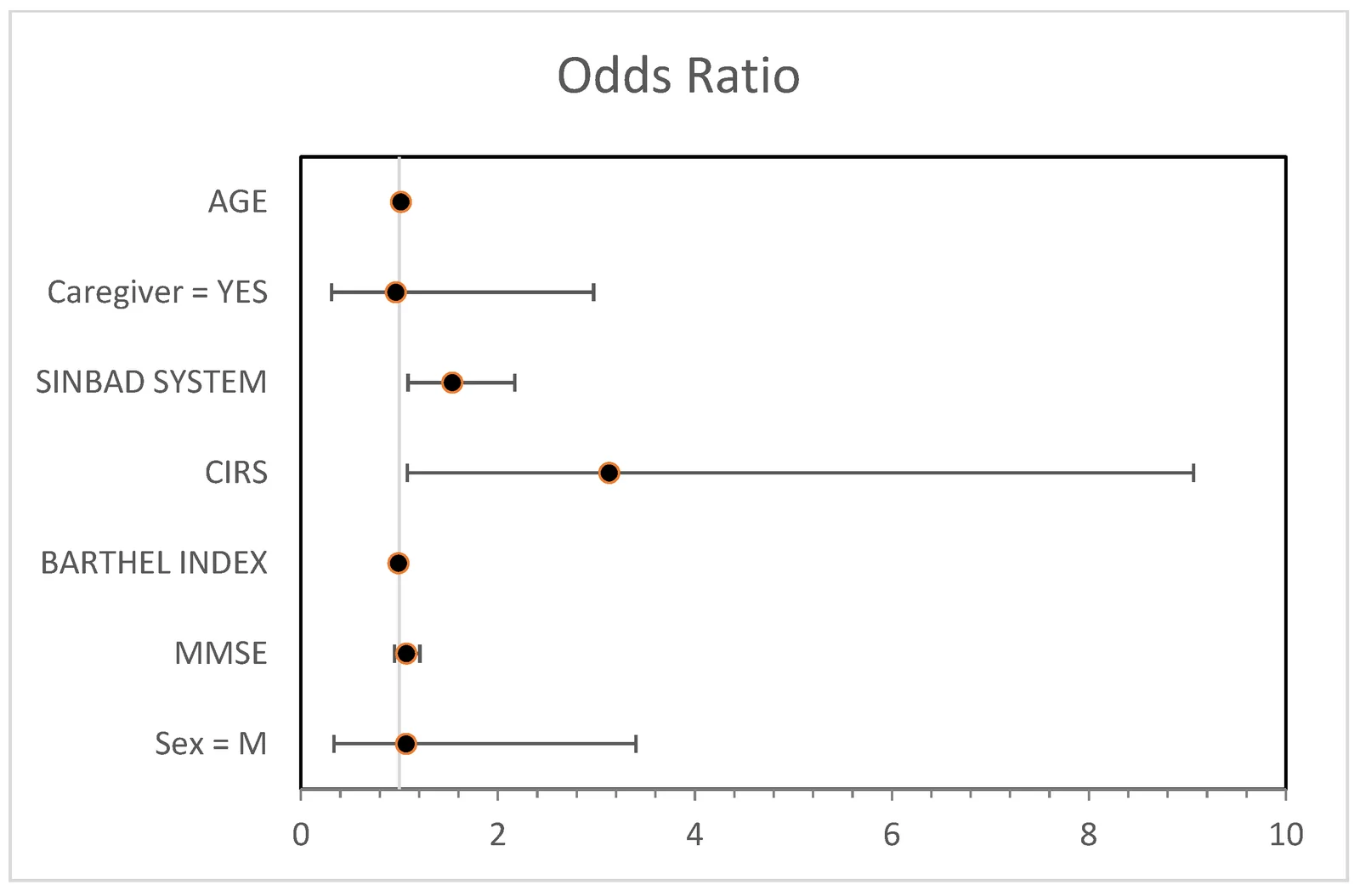
Representation of the ORs and confidence intervals of the multivariable model for the “minor amputation” outcome.

**Table 1:** Components of multidimensional complexity.

COMPLEXITY	Clinical Complexity	Presence of a single serious physical and/or mental pathology or presence of several comorbidities with severe impact on diagnosis and prognosis, with serious implications for clinical care [24].
	Nursing Complexity	Presence of physical or mental disabilities that cause non-self-sufficiency in ADL and IADL and that require support from others and/or the use of physical devices. It provides a measure of the intensity of care, i.e., the nursing workload provided to the patient [25].
	Socioeconomic Complexity (Environmental Complexity)	Presence of critical environmental issues related to the family nucleus, place of life, social relationships, and economic and working environment [26].

**Table 2:** Complexity assessment tools.

CIRS (Cumulative Illness Rating Scale)	Evaluates clinical complexity using the 13-item Severity Index obtained as the average of the scores of all categories, excluding the “Psychiatric and behavioral” category (range of values between 1 and 5) [31].
Barthel Index	Evaluates autonomy in basic activities of daily living (ADL), with a range from 0 (total dependence) to 100 (total independence) [32].
Lawton–Brody Scale	Evaluates autonomy in instrumental activities of daily living (IADL), with a range from 0 (total dependence) to 8 (total independence) [33].
MMSE (Mini-Mental State Examination)	Evaluates intellectual efficiency disorders and the presence of cognitive impairment (range of 0–30, corrected for years of schooling if the patient is over 65). A score ≥26 indicates preserved cognitive status, a score of 25 indicates a borderline level, and a score ≤24 indicates the presence of cognitive impairment [34].
SINBAD System	Tool for amputation risk stratification by evaluating site (Site), presence of valid peripheral pulses (Ischemia) and sensory neuropathy (Neuropathy), clinical signs of infection (Bacterial infection), ulcer area (Area), and depth of ulcer (Depth): a higher score expresses a greater risk of amputation (range of 0–6) [35].
MUST (Malnutrition Universal Screening Tool)	Evaluates nutritional status and risk of malnutrition. It considers BMI, unintentional weight loss and the impact of acute illness on caloric intake reduction (range 0–3). A score ≥2 indicates high risk of malnutrition; a score of 1 indicates medium risk of malnutrition; and a score of 0 indicates low risk of malnutrition [36].
ONAS Scale (Italian National Association of Social Workers Scale)	This scale studies social complexity through the evaluation of 4 areas in the patient’s social life (economic, housing, family, and activated services/benefits) with a range of 40–400 and identification of 3 levels of complexity (40–140: low complexity, 150–270: medium complexity, and 280–400: high complexity.

**Table 3:** Main characteristics of population.

	Total 115 (100%)		Males 84 (73%)		Females 31 (27%)		Inpatients 74 (64.3%)		Outpatients 41 (35.7%)	
Mean age	70.6		70.8		70		70		71	
Amputation	43 (37.4%)		35 (41.7%)		8 (25.8%)		41 (55.4%)		2 (4.9%)	
Type of Amputation	Major 5 (4.4%)	Minor 38 (33%)	Major 4 (4.8%)	Minor 31 (36.9%)	Major 1 (3.2%)	Minor 7 (22.6%)	Major 5 (6.8%)	Minor 36 (48.6%)	Major 0 (0%)	Minor 2 (4.9%)
No Amputation	72 (62.6%)		49 (58.3%)		23 (74.2%)		33 (44.6%)		39 (95.1%)	
Revascularization	51 (44.3%)		44 (52.4%)		7 (22.6%)		49 (66.2%)		2 (4.9%)	
Type of Revascularization	PTA 45 (39.1%)	Bypass 6 (5.2%)	PTA 40 (47.6%)	Bypass 4 (4.8%)	PTA 5 (16.1%)	Bypass 2 (6.5%)	PTA 43 (58.1%)	Bypass 6 (8.1%)	PTA 2 (4.9%)	Bypass 0 (0%)
No Revascularization	64 (55.7%)		40 (47.6%)		24 (77.4%)		25 (33.8%)		39 (95.1%)	
Caregiver	78 (70% of 110)		53 (67% of 79)		25 (80% of 31)		51 (71.8% of 71)		27 (69.2% of 39)	
No Caregiver	32 (30% of 110)		26 (33% of 79)		6 (20% of 31)		20 (28.2% of 71)		12 (30.8% of 39)	
Missing date Caregiver	5 (4% of 115)		5 (6% of 84)		0 (0%)		3 (4% of 74)		2 (4.9% of 41)	

**Table 4:** Comparison between group A (inpatients) and group B (outpatients).

	Inpatients (Group A)	Outpatients (Group B)
SINBAD system	4 (sd ± 1.5)	3 (sd ± 1.73)
CIRS (severity index)	2.23 (sd ± 0.5)	1.86 (sd ± 0.5)
ADLs (Barthel index)	86.7 (sd ± 21.7)	83.6 (sd ± 22.4)
IADLs (Lawton–Brody scale)	5.6 (sd ± 2.6)	4.8 (sd ± 2.7)
MMSE	24.3 (sd ± 4.5)	24.5 (sd ± 5.1)
Caregivers (%)	72%	69%
Malnutrition (MUST)	0.8 ≈ moderate risk	0.2 ≈ low risk
Socioeconomic complexity (ONAS)	142.6 = moderate risk	186.5 = moderate risk
Revascularization (%)	66%	4.9%
Amputation (%)	55.4%	4.9%
Major amputation	6.8%	0%
Minor amputation	48.6%	4.9%

**Table 5:** Multivariate associations between patient characteristics and minor amputation. Adjusted odds ratios were obtained through multivariate logistic regression model.

Characteristic	Adj. OR	95% CI	*p*-Value	n Analyzed
MMSE	1.07	0.95–1.21	0.270	97
BARTHEL INDEX	0.99	0.96–1.02	0.444	97
CIRS	3.13	1.08–9.06	0.035	97
SINBAD SCORE	1.54	1.09–2.17	0.015	97
AGE	1.01	0.97–1.06	0.533	97
SEX	1.07	0.34–3.40	0.912	97
CAREGIVER “YES”	0.96	0.31–2.97	0.947	97

**Table 6:** CIRS additional predictive information. The inclusion of CIRS produced a significant increment in multivariable model goodness of fit (LRT *p*-value, 0.023).

Model *	BIC	AUC	HLT *p*-Value	LRT *p*-Value
CIRS excluded	156.25	0.796	0.719	-
CIRS included	155.63	0.825	0.956	0.023

* Both models compared included the following as covariates: sex, MMSE, Barthel index, SINBAD, caregiver presence and age. Abbreviations: BIC: Bayesian information criterion; LRT: Likelihood ratio test; AUC: Area under the ROC curve; HLT: Hosmer–Lemeshow test.

## Data Availability

The original contributions presented in this study are included in the article. Further inquiries can be directed to the corresponding author.

## Abbreviations

The following abbreviations are used in this manuscript:

ADLs	Activities of Daily Living
CIRS	Cumulative Illness Rating Scale
DFS	Diabetic Foot Syndrome
IADLs	Instrumental Activities of Daily Living
MMSE	Mini-Mental State Examination
MUST	Malnutrition Universal Screening Tool
ONAS	Ordine Nazionale Assistenti Sociali (Italian National Association of Social Workers)
PAD	Peripheral Arterial Disease
ICP	Integrated Care Pathway
PTA	Percutaneous Transluminal Angioplasty
